# Genomic characterization and evolution analysis of peste des petits ruminants virus in China from 2007 to 2024

**DOI:** 10.3389/fmicb.2025.1697536

**Published:** 2025-11-21

**Authors:** Jingyue Bao, Ruiying Jiang, Shujuan Wang, Qinghua Wang, Jiao Xu, Yunling Zhao, Yutian Liu, Lin Li, Xiaohua Wang, Zhiliang Wang

**Affiliations:** 1 China Animal Health and Epidemiology Center, Qingdao, China; 2 College of Wildlife and Protected Area, Northeast Forestry University, Harbin, China

**Keywords:** peste des petits ruminants virus, lineage IV, genomic epidemiology, evolution, phylogenetic analysis

## Abstract

Peste des petits ruminants (PPR) is a highly contagious and lethal disease, primarily affecting sheep, goats, and wild small ruminants. Genomic characterization and evolutionary analysis of the circulating PPR virus (PPRV) strains can provide valuable information for the implementation of efficient control measures. In this study, we generated 28 novel PPRV full-length genome sequences from clinical samples collected during 28 PPR outbreaks in livestock and wildlife hosts across 15 provinces in China from 2014 to 2021. These genome sequences were compared with 135 published PPRV genome sequences, 37 of which were collected from China. The evolutionary rate of the PPRV genome was estimated to be 6.70 × 10^−4^ nucleotide substitutions per site per year. The predominant PPRV lineage IV can be divided into seven clades, demonstrating the temporal and spatial correlation. PPRV in China from 2007 to 2008 and 2013 to 2024 were grouped into two distinct genetic clades in lineage IV, indicating two independent incursions of the disease in 2007 and 2013. The PPRV in China from 2013 to 2024 shared a common ancestor with a strain from the UAE and evolved into four distinct genetic clusters, which did not exhibit distinct temporal, spatial, and host correlation. We used PPRV/XJYL/2013 as the reference genome, and 997 single-nucleotide variations (SNVs) were identified in PPRV genomes in China from 2013 to 2024. A total of five single-nucleotide variations, located in the 3′ leader, 5′ untranslated region (UTR) of the F gene, H cds, and L cds, were identified as anchor mutations, which defined the genetic clusters of PPRV during this period. One site in the H gene and five sites in the L gene were identified under positive selection. Our study provides significant insights into the molecular epidemiology, evolution, and transmission of PPRV, which will support the development of effective strategies for PPR control and eradication.

## 1 Introduction

Peste des petits ruminants (PPR) is a highly contagious and lethal viral disease that infects sheep, goats, and wild small ruminants. PPR was first reported in Côte d’Ivoire in 1942. Since then, it has been reported in Africa, Asia, the Middle East, and, recently, in Europe (Donduashvili et al., 2018). PPR has severely impacted the development of the sustainable small ruminant industry, seriously affecting the livelihoods, food security, and nutrition of small-scale farmers and pastoralists. The annual global impacts of PPR have been estimated between US$1.4 billion and US$2.1 billion (Jones et al., 2016). The Food and Agriculture Organization of the United Nations (FAO) and the World Organisation for Animal Health (WOAH) have officially launched a global program to eradicate PPR by 2030 (FAO, WOAH, 2022; OIE, FAO, 2015).

PPR is caused by the PPR virus (PPRV), a member of the genus *Morbillivirus* in the family *Paramyxoviridae*. The genome of PPRV is a non-segmented negative-strand RNA molecule of length 15,948 or 15,954 nucleotides (nt), encoding the nucleoprotein (N), phosphoprotein (P), matrix protein (M), fusion protein (F), hemagglutinin protein (H), and polymerase or large protein (L) (Bailey et al., 2005; Bao et al., 2014). The P transcription unit also encodes two non-structural proteins: C and V. Phylogenetic analysis based on the partial sequence of the N gene (255 nt region, nucleotide site 1,360–1,614 of the PPRV genome) or the F gene (322 nt region, nucleotide site 5,779–6,100 of the PPRV genome) has classified PPRV into four lineages (Kwiatek et al., 2007; Shaila et al., 1996). Lineages I and II contained viruses from Africa; lineage III included viruses from the Arabian Peninsula and Africa; and lineage IV viruses were obtained from Asia and the Middle East & Africa. In recent years, lineage IV viruses have dominated Africa, Asia, the Middle East, and Eastern European regions. However, why lineage IV is more widespread than the others remains unclear.

Genomic epidemiological analyses based on full-genome sequences of the virus have provided valuable information for interpreting field epidemiology data and implementing efficient control measures (Guo et al., 2025; Nyathi et al., 2024). The evolutionary dynamics of PPRV have been studied to understand the origin of PPRV lineages (Mahapatra et al., 2021; Muniraju et al., 2014). Genomic evolutionary analysis of PPRV in wildlife highlighted its dynamics at the livestock-wildlife interface in Mongolia (Benfield et al., 2021). Comparative genomic analysis revealed varying evolutionary dynamics between lineages II and IV (Courcelle et al., 2024). In addition, genomic epidemiology analysis has been used to trace the evolution of PPRV in China from 2013 to 2014, in Israel from 1993 to 2014, and in Bangladesh from 2008 to 2020 (Bao et al., 2017; Clarke et al., 2017; Nooruzzaman et al., 2021). Large-scale, detailed, and up-to-date studies on the genomic epidemiology and evolutionary dynamics of PPRV lineage IV are urgently required to address the challenges hindering global PPR eradication efforts.

In China, the first incursion of PPR was recorded in 2007 in the Xizang province, which persisted until 2010 and faded thereafter (Wang et al., 2009). Molecular phylogenetic analysis of partial N and F gene sequences showed that the 2007 PPRV Chinese strains belonged to lineage IV, forming a cluster with the strains collected in India, Nepal, Bhutan, and Bangladesh. PPR was reintroduced in the Xinjiang province of China in 2013, spreading across the country. It has sporadically resurfaced in the country ever since (Banyard et al., 2014; Wu et al., 2016). Molecular phylogenetic analysis shows that the PPRV strains detected in China in 2013 clustered within a distinct clade of lineage IV with strains obtained from Tajikistan, Kazakhstan, and Mongolia. In-depth evolutionary analysis of full-length genome sequences has classified 25 PPRV strains from China (2013–2014) into five distinct clusters (Bao et al., 2017). A few genome sequences have been characterized for PPRV strains collected in China from 2018 to 2024 (Li et al., 2019; Li et al., 2021; Xu et al., 2024). However, the evolutionary dynamics of PPRV lineage IV from 2014 to 2024 remain unknown.

PPRV infection of wildlife in China has been reported in bharal (*Pseudois nayaur*) (Bao et al., 2011), ibex (*Capra ibex*) (Li et al., 2017; Xia et al., 2016; Zhu et al., 2016), argali sheep (*Ovis ammon*) (Li et al., 2017), goitered gazelle (*Gazella subgutturosa*) (Li et al., 2017), and Przewalski’s gazelle (*Procapra przewalskii*) (Li et al., 2019). The partial N or F gene sequences or genome sequences of PPRV strains from these wildlife species have also been reported in the country. However, the evolutionary dynamics of PPRV at the livestock–wildlife interface in the country are yet to be studied.

In this study, we generated 28 novel PPRV full-length genome sequences from clinical samples collected during 28 PPR outbreaks in livestock and wildlife hosts in China from 2014 to 2021. We assessed the genetic diversity of these sequences and other published PPRV genomes obtained in the country. In addition, we conducted phylogenetic analysis to study the evolutionary dynamics of PPRV in livestock and at the livestock–wildlife interface between 2007 and 2024. Moreover, we investigated the single-nucleotide variations (SNVs) in PPRV genomes to identify the anchor mutations that define the genetic clusters of PPRV in China.

## 2 Materials and methods

### 2.1 Genome sequencing

PPRV genome sequences were obtained from tissue samples of infected animals, collected from 28 PPR outbreaks in 15 provinces across China from 2014 to 2021. Viral RNA was extracted and used directly for viral genome sequencing. A total of 14 pairs of oligonucleotide primers were used to amplify the 14 overlapping fragments by reverse transcription-polymerase chain reaction (PCR), as previously described (Bao et al., 2014). The PCR products were purified and sequenced with an ABI 3730XL genome sequencer (Applied Biosystems, USA).

### 2.2 Genome sequences alignment and recombination analysis

All PPRV genome sequences in the NCBI Nucleotide database were obtained on 22 July 2024. The detailed information for each sequence, including country, host, and collection date, was extracted and added to the dataset. Vaccine strains were not included in this study. The MAFFT software (version 7.475) was used to perform genome sequence alignment and determine the nucleotide sequence similarity (Katoh et al., 2002). The RDP software (version 4.101) was used to analyze recombination with seven different recombination detection methods (RDP, GENECONV, BootScan, MaxChi, Chimaera, SiScan, and 3Seq) (Martin et al., 2015). The window size was set to 200, with all other settings at default values. Events identified by at least five out of the seven different detection algorithms with a *p*-value cutoff of 0.01 were considered true recombinant events. The TempEst software was used to assess the temporal signal of the sequence dataset (Rambaut et al., 2016).

### 2.3 Phylogenetic analysis

Time-scaled phylogenies of PPRV were reconstructed using the Bayesian Markov Chain Monte Carlo (MCMC) methods in the BEAST package v1.8.2 (Drummond et al., 2012). JModelTest software (v2.1.10) was used to select the most appropriate substitution models (Darriba et al., 2012). The GTR + I + G substitution model under a lognormal uncorrelated relaxed clock model with a coalescent constant size model was used to analyze the dataset. The MCMC chains of 4 × 10^8^ generations were run for the analysis and sampled every 40,000 generations. The Maximum-clade credibility (MCC) trees were generated using TreeAnnotator v1.10.4 with a burn-in rate of 10%. The convergence was examined using Tracer software v1.7, where all parameters yielded an effective sample size (ESS) greater than 200. FigTree v1.4.2 was used to summarize the phylogenies.

### 2.4 Phylogenetic network analysis

The genome PPRV/XJYL/2013, which was collected during the first outbreak of PPR in Xinjiang province, China, in November 2013, was selected as the reference genome. All the single-nucleotide variations (SNVs) along the genome sequences of PPRV from 2013 to 2024 were extracted from the aligned dataset and listed in a matrix. All the extracted SNVs were used to conduct phylogenetic network analysis with the Network software (version 10.2) (Bandelt et al., 1999). The median-joining network and the Steiner algorithms were applied. The network plot was manually adjusted for better visualization.

### 2.5 Detection of selection pressures

Positive selection in the dataset was detected using single likelihood ancestor counting (SLAC), fixed effects likelihood (FEL), mixed effects model of evolution (MEME), and fast unconstrained Bayesian approximation (FUBAR) with the Hyphy software (Kosakovsky Pond and Frost, 2005). When identified by at least two algorithms, sites were considered under positive selection.

## 3 Results

### 3.1 The genomic diversity of PPRV in China

**Figure 1:**
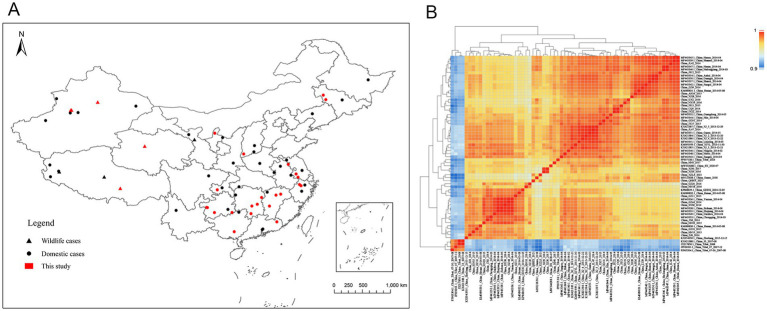
Geographic distribution and sequence similarity of PPRV genome sequences in China. **(A)** Location of infected farms where the PPR viruses were collected and sequenced. Domestic cases are represented by circles, wildlife cases are represented by triangles, and red represents cases sequenced in this study. **(B)** Sequence similarity matrix plot of PPRV genome sequences in China. The level of identity of pairwise genome sequences is indicated by different colors. Dark red represents 100% identity, and blue represents lower identity.

For this study, we obtained 28 PPRV full-length genome sequences from 28 outbreaks in 15 Chinese provinces from April 2014 to February 2021 (Supplementary Table S1). These genomes were collected from sheep or goats (23 genomes), bharals (2), goitered gazelles (2), or Siberian ibex (1). All the sequences shared an identical length of 15,954 nt. To investigate the genomic diversity of PPRV in China, these genomes were combined with 30 Chinese PPRV genomes previously obtained in our laboratory and 7 Chinese PPRV genomes submitted to the NCBI Nucleotide database by other laboratories (Supplementary Table S1). In total, 65 PPRV full-length genome sequences, representing 65 PPRV outbreaks in 23 provinces in China from 2007 to 2024, were used for further analysis (Figure 1A).

Pairwise sequence similarity was calculated to investigate the genome-wide diversity of PPRV in China. The overall genome-wide sequence similarity of PPRV strains from 2007 to 2024 varied by 96.5 to 100% (Figure 1B). As expected, the PPRV strains obtained from China during 2007–2008 displayed a lower sequence similarity (96.5–97.3%) to the strains collected between 2013 and 2024. The sequence similarity among PPRV strains isolated between 2007 and 2008 ranged from 99.8 to 100% and that among strains isolated between 2013 and 2024 ranged from 98.5 to 100%.

### 3.2 Phylogenetic analysis of PPRV in China from 2007 to 2024

For phylogenetic analysis, 106 PPRV field strain genome sequences from other countries were retrieved from the NCBI nucleotide database on 22 July 2024. In total, eight sequences (KR828814.1, KJ867541.1, KY967609.1, OK274213.1, OR286481.1, KY967608.1, MW960272.1, and KR261805.1) with signatures of recombination by at least five of the seven different detection algorithms were extracted (*p* < 0.01) (Supplementary Table S2). The curated dataset of 98 PPRV strains was combined with 65 PPRV genomes from China to create a set of 163 PPRV genome sequences for further phylogenetic analysis (Supplementary Table S1). These genomes were obtained from 10 countries in Asia (107 genomes) and 21 countries in Africa (56 genomes) from 1969 to 2024 (Supplementary Figure S1). Sheep or goats (144 genomes), bharals (4), goitered gazelles (3), Mongolian saiga (2), Siberian ibex (2), Barbary sheep (1), wild goat (1), Nubian ibex (1), dorcas gazelle (1), mountain gazelle (1), and Przewalski’s gazelle (1) acted as the host for these PPRV genomes.

**Figure 2:**
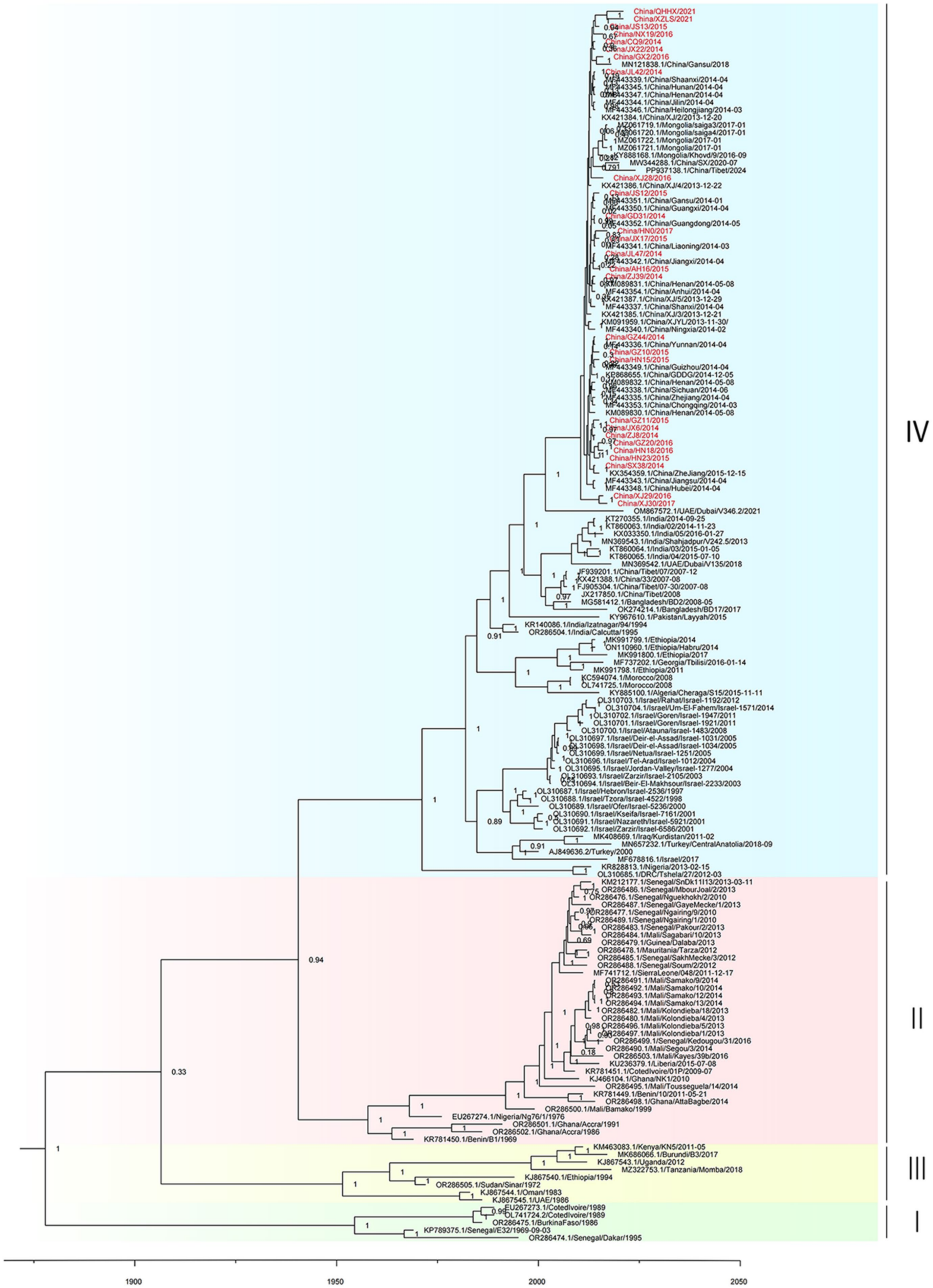
Time-resolved phylogenetic tree based on PPRV genome sequences using Bayesian MCMC analysis. The tree was estimated using the GTR + I + G substitution model under a lognormal uncorrelated relaxed clock model with a coalescent constant size model. The scale bar indicates time in years. The posterior probability is shown at each node. Sequences obtained in this study are shown in red.

**Figure 3:**
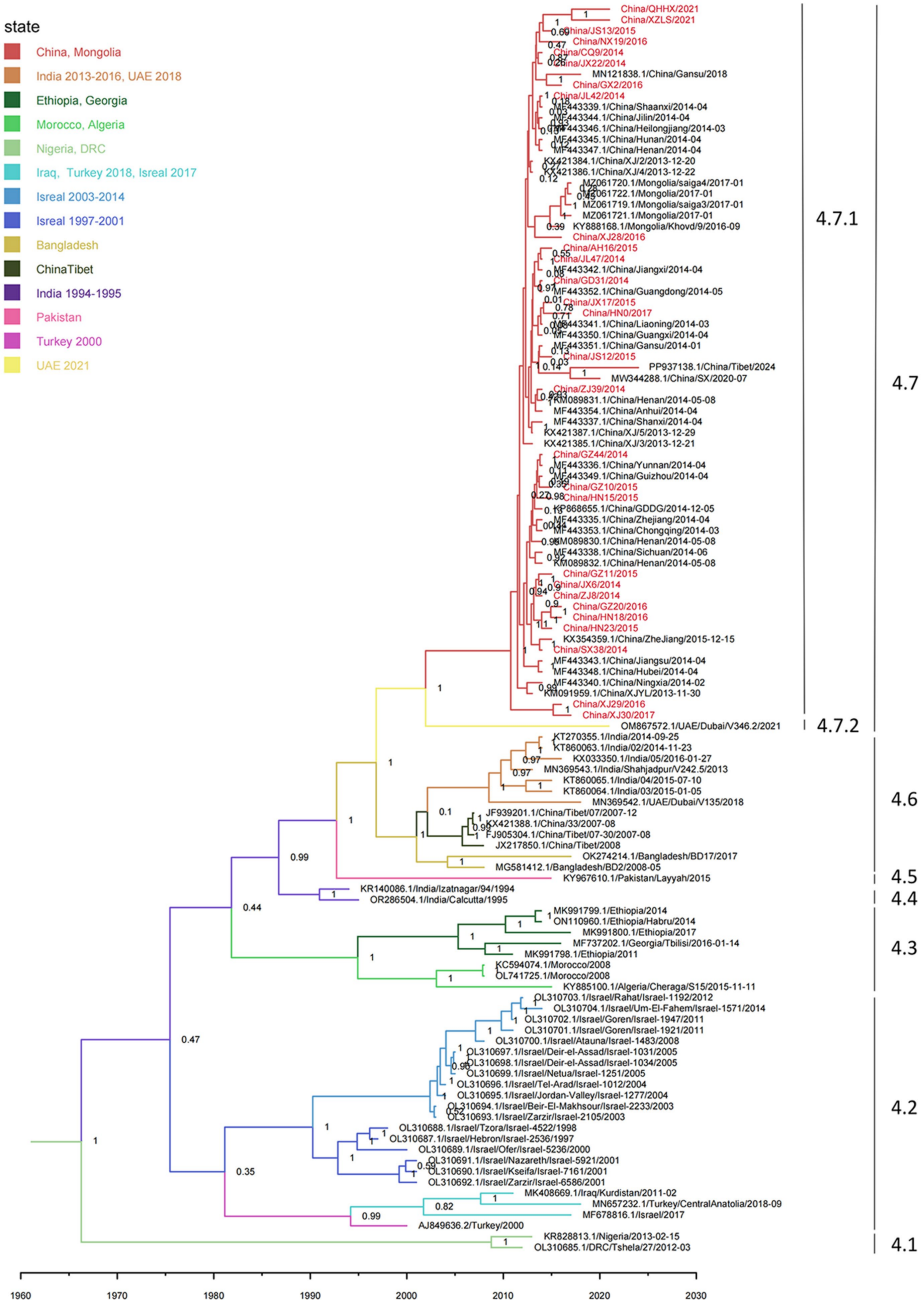
Time-resolved phylogenetic tree based on PPRV lineage IV genome sequences using Bayesian MCMC analysis. The tree was estimated using the GTR + I + G substitution model under a lognormal uncorrelated relaxed clock model with a coalescent constant size model. The scale bar indicates time in years. The branches are color-coded by country as inferred using discrete trait analyses. The posterior probability is shown at each node. Sequences obtained in this study are shown in red.

Root-to-tip regression analysis was employed to assess the temporal signal from the alignment of 163 PPRV genome sequences sampled between 1969 and 2024. The analysis highlighted a strong association between genetic distance and sampling date, with a correlation coefficient of 0.8812 and a coefficient of determination (R^2^) of 0.7765 (Supplementary Figure S2). The time-resolved phylogenetic analysis of 163 PPRV genome sequences indicated that their mean evolutionary rate was estimated to be 6.70 × 10^−4^ nucleotide substitutions per site per year (95% highest posterior density [95% HPD], 5.63 × 10^−4^; 7.87 × 10^−4^; Figure 2). In addition, the time-resolved phylogenetic analysis of 115 PPRV lineage IV genome sequences indicated their mean evolutionary rate was 7.54 × 10^−4^ nucleotide substitutions per site per year (95% highest posterior density [95% HPD], 5.98 × 10^−4^; 9.12 × 10^−4^). The root age of all the lineage IV PPRV genomes was estimated to be December 1966 (95% HPD, March 1937–May 1984). The 115 PPRV lineage IV strains clustered into seven clades showing temporal and spatial correlation, which were designated clades 4.1–4.7 (Figure 3). The time to the most recent common ancestry (TMRCA) of the different clades of lineage IV PPRV ranged from April 1982 to April 2008 (Table 1). PPRV strains from 2007 to 2008 and 2013 to 2024 in China were grouped into clade 4.6 and clade 4.7, respectively.

**Table 1:** Estimated TMRCA for different clades of the lineage IV PPRV phylogeny.

Set description	TMRCA	95% HPD Interval	Number of sequences	Time period
Lineage IV	December 1966	March 1937–May 1984	115	1994–2024
Clade 4.1	April 2008	May 2023–April 2001	2	2012–2013
Clade 4.2	April 1982	January 1972–February 1990	22	1997–2018
Clade 4.3	December 1993	February1982-February 2002	8	2008–2017
Clade 4.4	June 1990	March 1993–May 1986	2	1994–1995
Clade 4.5	/	/	1	2015
Clade 4.6	April 2001	April 1997–May 2004	13	2007–2018
Clade 4.7	June 2002	June 1996–March 2008	67	2013–2024

Monophyletic grouping of all sequences in clade 4.6 was strongly supported (100% posterior probability). The TMRCA of clade 4.6 was estimated to be April 2001 (95% HPD: April 1997–May 2004). In clade 4.6, all four PPRV genomes in China (2007–2008) shared a single origin and had a common ancestor with a cluster of two PPRV strains collected from Bangladesh between 2008 and 2017. Clade 4.6 also included a cluster of six strains from India (2014–2016) and a singleton of a strain from the UAE in 2018. Clade 4.6 was inferred to have originated in Bangladesh, with strong support (100% root state posterior probability). In addition, monophyletic grouping of all the sequences in clade 4.7 was strongly supported (100% posterior probability). The TMRCA of clade 4.7 was dated to June 2002 (95% HPD: February 1996–March 2008). In clade 4.7, all the PPRV genomes in China (2013–2024) and Mongolia (2016–2017) shared a single origin (sub-clade 4.7.1) and a common ancestor with a PPRV strain from the UAE in 2021 (sub-clade 4.7.2). The latest divergence of PPRV sub-clade 4.7.2 was 7.5 years earlier than the TMRCA of sub-clade 4.7.1. The country of origin for clade 4.7 was inferred UAE with high support (100% root state posterior probability).

### 3.3 Subgroup divergence of PPRV in China from 2013 to 2024

**Figure 4:**
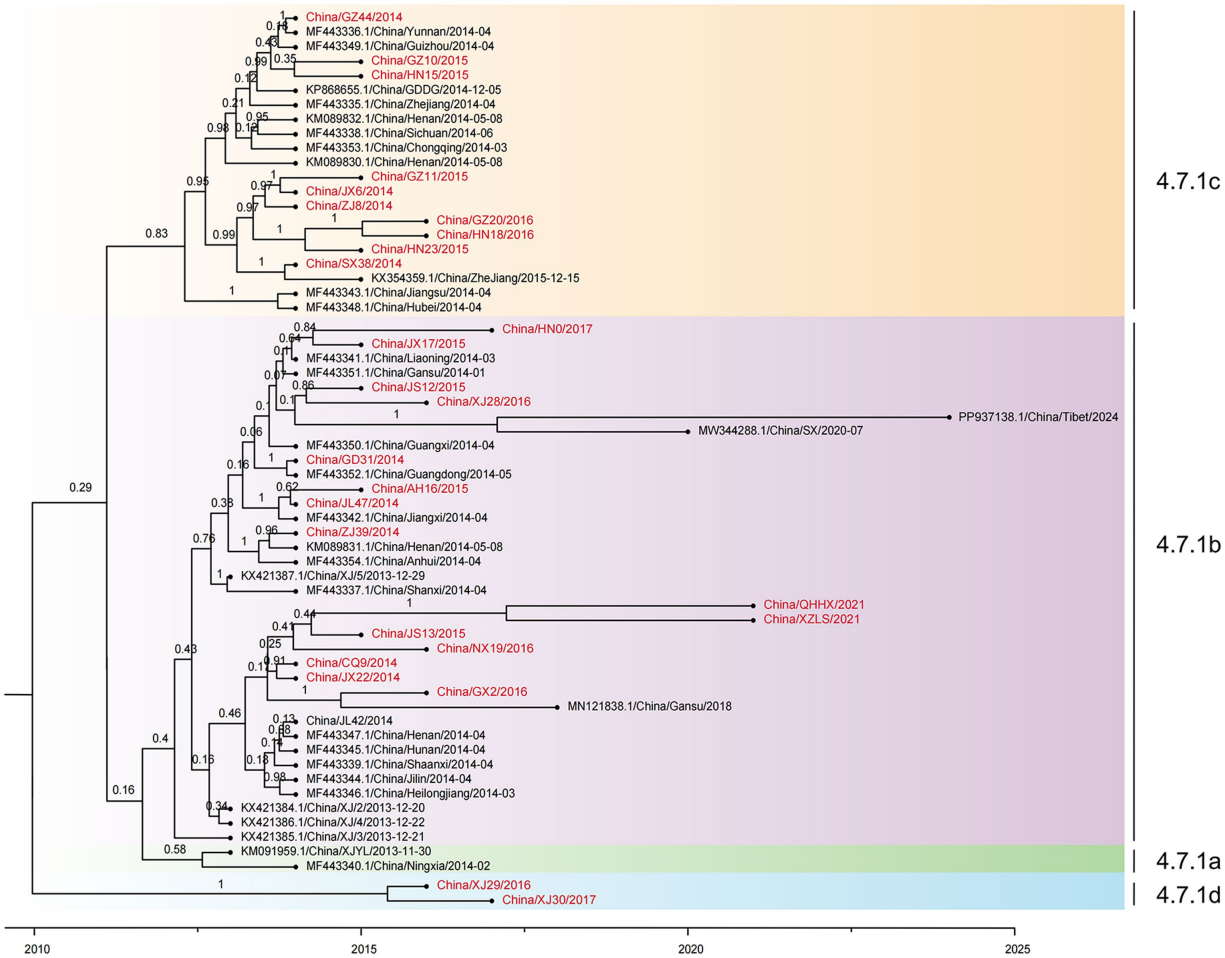
Time-resolved phylogenetic tree based on 61 PPRV genome sequences in China from 2013 to 2024 using Bayesian MCMC analysis. The tree was estimated using the GTR + I + G substitution model under a lognormal uncorrelated relaxed clock model with a coalescent constant size model. The scale bar indicates time in years. The posterior probability is shown at each node. Sequences obtained in this study are shown in red.

Time-resolved phylogenetic analysis was performed for all 61 PPRV genomes from China (2013–2024). Sequences collected from 23 different provinces in China from 2013 to 2024 were divided into four distinct clusters, designated cluster 4.7.1a-4.7.1d (Figure 4): cluster 4.7.1a contained two early strains, China/XJLY/2013 and China/NX/2014; cluster 4.7.1b included 36 strains collected from 2013 to 2024; and cluster 4.7.1c included 21 strains collected from 2014 to 2016. The monophyletic grouping of cluster 4.7.1d was strongly supported. It contained two PPRV strains collected from goitered gazelle in Xinjiang province between March 2016 and March 2017 (99.92% posterior probability).

**Figure 5:**
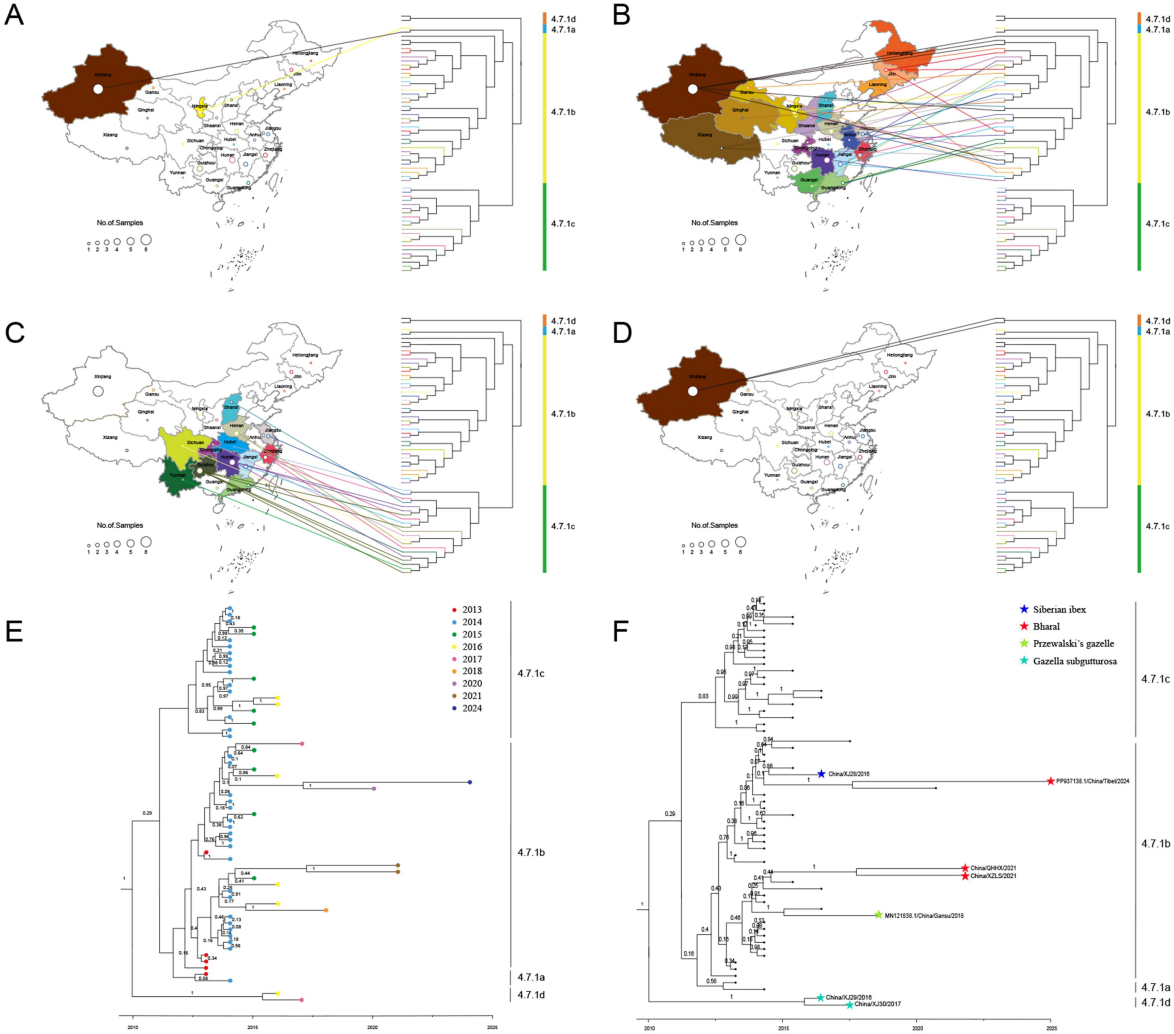
The spatiotemporal and livestock–wildlife interface distribution of PPRV sequences in China during 2013–2024. The geographical distribution of cluster 4.7.1a **(A)**, cluster 4.7.1b **(B)**, cluster 4.7.1c **(C)**, and cluster 4.7.1d **(D)**. The temporal distribution **(E)** and livestock–wildlife interface distribution **(F)** of PPRV sequences in China during 2013–2024.

All the PPRV strains in clusters 4.7.1a, 4.7.1b, and 4.7.1c shared a single origin, which dated back to January 2011 (95% HPD, May 2008; March 2012). However, the latest divergence of PPRV from cluster 4.7.1d to the common ancestor of clusters 4.7.1a, 4.7.1b, and 4.7.1c dated back to December 2009 (95% HPD, January 2002; March 2012), which was 13 months earlier than the TMRCA of the common ancestor of clusters 4.7.1a, 4.7.1b, and 4.7.1c. The cluster 4.7.1b strains emerged in 2013 and were sampled every year until 2024, except 2019 and 2021 (Figure 5E). The cluster 4.7.1c strains emerged in 2014 and faded after 2016. It is indicated that PPRV cluster 4.7.1b has been the temporally dominant cluster in China. PPRV strains from different years are interspersed in cluster 4.7.1b or cluster 4.7.1c, with no distinct temporal correlation among the strains in each cluster.

The geographical distribution of PPRV from China (2013–2024) was further investigated. Clusters 4.7.1a and 4.7.1d strains were collected only from Xinjiang province (Figures 5A–D). Cluster 4.7.1b strains were found to be widely distributed across 19 provinces, covering all seven geographic regions in the country (Figure 5B). Cluster 4.7.1c strains were collected from 12 provinces across five geographic regions, with no sample from Northeast China or Northwest China (Figure 5C). Samples from different provinces intermixed in clusters 4.7.1b and 4.7.1c, with no sign of within-province or within-region clustering. For instance, a strain collected in Xizang province in 2024 was most closely related to a strain collected in Shaanxi province in 2020.

The distribution of PPRV sequences at the livestock-wildlife interface was further investigated. Except for the monophyletic grouping of two strains from the goitered gazelle in Xinjiang into cluster 4.7.1d, all the other 5 wildlife strains were grouped into cluster 4.7.1b (Figure 5F). In cluster 4.7.1b, it was of interest that a strain from bharal in Xizang in 2021 was grouped with a strain from bharal in Qinghai in 2021 with strong support (100% posterior probability). However, the other 3 sequences from wildlife were interspersed with those from sheep or goats. For instance, a strain from Siberian ibex in Xinjiang in 2016 was grouped with a strain from a goat in Jiangsu in 2015 with strong support. A strain from Przewalski’s gazelle in Gansu in 2018 was grouped with a strain from a goat in Guangxi in 2016 with strong support. The PPRV China/XZ/2024 from bharal in Xizang in 2024 was most closely related to a strain from a goat sampled in Shaanxi in July 2020 (100% posterior probability).

### 3.4 Identification of anchor mutations in PPRV genomes collected in China from 2013 to 2024

**Figure 6:**
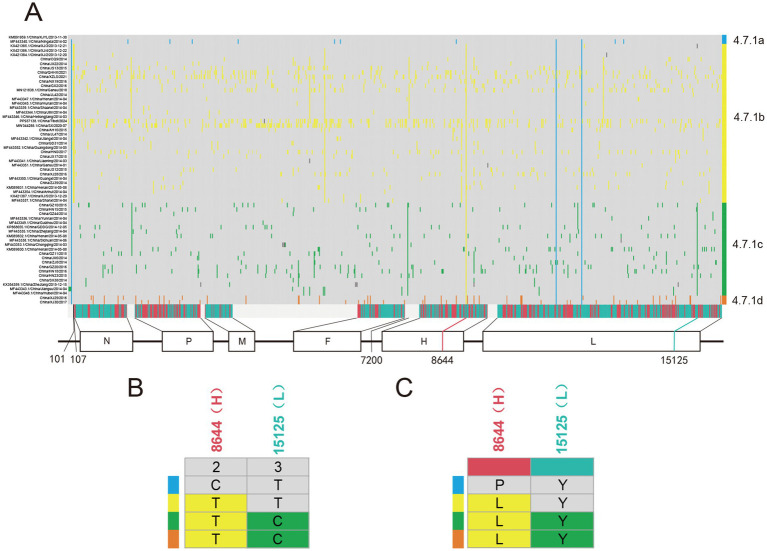
Pattern of PPRV single-nucleotide variations (SNVs) distribution in China from 2013 to 2024. **(A)** Distribution of SNVs in PPRV genomes in China from 2013 to 2024. The strain name is shown at the left, indicating one strain per row. Grey blocks indicated sequence identity with the reference genome PPRV/XJYL/2013 sequence. The last row shows the type of mutation (grey, intergenic; green, synonymous; and red, non-synonymous), with the gene location indicated below. Cluster assignment is shown at the right by color. **(B)** Distribution of five anchor SNVs in different clusters of PPRV genomes. The top row shows the codon position of SNVs with the gene location indicated above. **(C)** Distribution of amino acid changes of anchor SNVs in different clusters of PPRV genomes. The top row shows the type of mutation (green, synonymous; and red, non-synonymous), with the gene location indicated above.

To trace the occurrence and possible fixation of mutations during the evolution of PPRV in China from 2013 to 2024, we investigated the single-nucleotide variations (SNVs) in PPRV genomes. A total of 997 SNVs were identified and listed in a matrix (Figure 6A). Five SNVs were identified as anchor mutations, which were fixed in all sequences in the corresponding clusters. The gene location and codon position of each anchor mutation were displayed (Figure 6B). The distribution of amino acid changes of each anchor mutation was also investigated (Figure 6C). Sequences in cluster 4.7.1a shared one non-synonymous mutation, C8644T (C1313T/Pro438Leu in H gene). Sequences in cluster 4.7.1b were distinguished from other clusters by two mutations (T101C and T107C) in the 3′ leader region of the genome. Cluster 4.7.1c was defined by one non-synonymous mutation (T15125C) in the L gene (T5832C/Tyr1944) and one mutation (A7200G) in the 5’ UTR region of the F gene.

**Figure 7:**
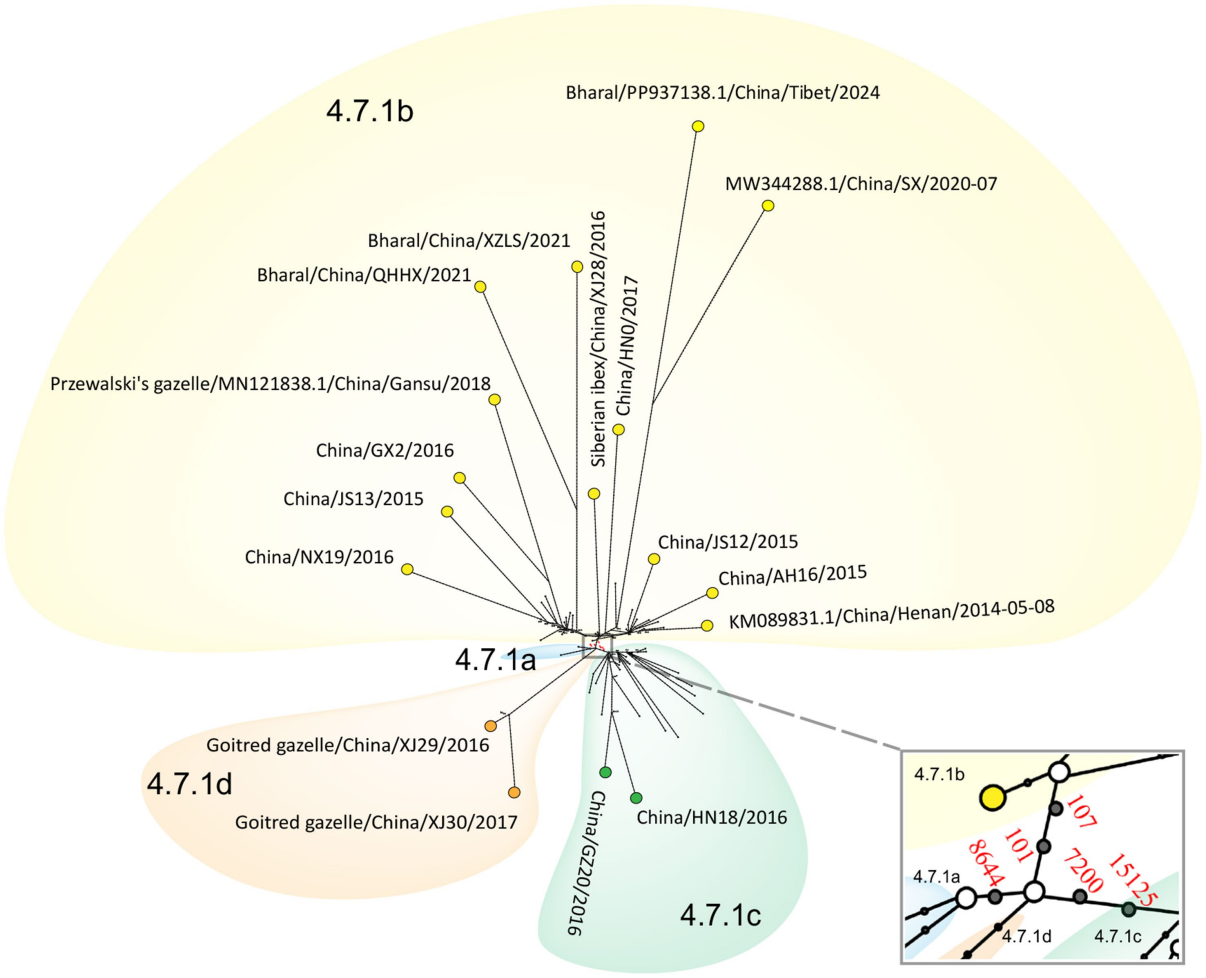
Phylogenetic network of PPRV genomes in China from 2013 to 2024. The network was constructed from the SNVs in the genome sequences. Circle nodes represented PPRV strains, which were proportional to the number of taxa. Each notch on the links represents a mutation event. Blank dots indicate putative ancestor nodes. Cluster assignment is shown by color. The median-joining network and the Steiner algorithms were used.

Median-joining phylogenetic network was constructed based on SNVs to explore the evolutionary relationship of PPRV in China from 2013 to 2024. In total, four clusters were identified mutationally branching from a single common node (Figure 7). The putative ancestor of cluster 4.7.1a was derived from the common node by one anchor mutation (C8644T), and the putative ancestor of cluster 4.7.1b was derived by two anchor mutations—T101C and T107C. Furthermore, the putative ancestor of cluster 4.7.1c was derived from the common node by two anchor mutations—A7200G and T15125C. Mutational branches in clusters 4.7.1b and 4.7.1c formed star-like radiation topology, indicating a single origin and the expansion of strains in the cluster. Expansion of long branches in cluster 4.7.1b suggested that it was the dominant circulating cluster in China in recent years.

### 3.5 Measure of selection pressures in lineage IV PPRV genomes

Site-specific selection pressures were measured as the ratio of non-synonymous (dN) to synonymous (dS) nucleotide substitutions per site to infer sites subjected to positive selection in the coding sequences of the H and L genes. Using a posterior probability of 0.95 for FUBAR, one site in the L gene was identified as under positive selection. Using the default threshold of significance of *p* < 0.1, one site in the H gene and five sites in the L gene were identified using the MEME and FEL methods. Amino acid 438 in the H gene was identified as under positive selection by both MEME and FEL methods (Supplementary Table S3).

## 4 Discussion

Our estimation of the evolutionary rate of 163 PPRV genomes collected between 1969 and 2024 is 6.70 × 10^−4^ nucleotide substitutions per site per year (95% HPD, 5.63 × 10^−4^; 7.87 × 10^−4^), which is consistent with previous studies (Supplementary Table S4) (Benfield et al., 2021; Clarke et al., 2017; Courcelle et al., 2024; Mahapatra et al., 2021). The evolutionary rate of 115 PPRV lineage IV genomes collected from Africa and Asia between 1994 and 2024 was estimated as 7.54 × 10^−4^ nucleotide substitutions per site per year (95% HPD, 5.98 × 10^−4^; 9.12 × 10^−4^), which is slightly faster than that of all other PPRV lineages. The widespread circulation of lineage IV PPRV in Africa and Asia, mass vaccination, large population of sheep and goats, and the inadequate biosecurity measures in the sheep and goat industries of Africa and Asia may have accelerated the evolution of the virus. The median TMRCA for lineage IV PPRV was estimated to be 1966 (95% HPD, March 1937–May 1984), which is equivalent to previous estimates (Supplementary Table S5) (Benfield et al., 2021; Clarke et al., 2017; Courcelle et al., 2024; Mahapatra et al., 2021).

This study supports that the PPRV clade 4.6 in China (2007–2008) shared a common origin with strains from India and Bangladesh, dating back to April 2001. However, we were unable to ascribe its country of origin due to a data gap for these countries, which are not represented by PPRV genome sequences or have a limited number of published PPRV genome sequences. Previous molecular phylogenetic analysis on N and F partial sequences also illustrated that Chinese PPRV strains from 2007 to 2008 were most closely related to the strains collected in India during 1999–2015, Nepal during 2005–2016, Bhutan in 2010, and Bangladesh during 2008–2017 (Kumar et al., 2014; Nooruzzaman et al., 2021). Among these countries, PPRV genome sequences from Nepal and Bhutan are not publicly available, and the Bangladesh PPRV genome sequence from 2001 to 2017 is unpublished. Moreover, the genome sequences of PPRV in India between 2001 and 2017 are limited. Prior studies demonstrated that sequencing a higher number of PPRV samples can dramatically improve the resolution of PPRV phylogenetic structure and enable finer molecular dating of PPRV (Bao et al., 2017). Information on other genome sequences of PPRV strains circulating in South Asia will likely improve our knowledge of the molecular epidemiology of PPRV clade 4.6 in the region. Despite the elimination of PPRV clade 4.6 in Tibet, China, after the last outbreak in 2010, understanding the evolutionary dynamics of PPRV in South Asia will help allocate the risk of re-incursion of PPRV clade 4.6 into China.

The study concluded that PPRV in China (2013–2024) clustered with five strains from Mongolia (2016–2017) and one strain from the UAE in 2021 into a distinct clade (clade 4.7), sharing a common ancestor dating back to 2002. The molecular phylogenetic analysis based on N and F partial sequences showed that the PPRV 2013–2024 strains in China were most closely related to strains obtained in Iran from 2014 to 2016, Pakistan from 2010 to 2014, Tajikistan in 2004, and Kazakhstan in 2014 (Kock et al., 2015; Marashi et al., 2017; Shahriari et al., 2019; Wu et al., 2016). It is well demonstrated that PPRV clade 4.7 has been circulating in the region since the 2000s. However, efforts to investigate the transmission route of PPRV clade 4.7 from the UAE to China were hindered by the lack of PPRV genome sequence in the Middle East and Central Asia. Knowledge of genome sequences of PPRV strains from Central Asia will improve our understanding of the transmission patterns of PPRV clade 4.7 in the region.

The 13-month time span between the divergence of cluster 4.7.1d and the common ancestor of the other 4.7.1 clusters suggests that their incursions are unlikely to have occurred in the same period. Previous studies have established that PPRV was first introduced into the Yili region of Xinjiang province in 2013 (Wu et al., 2016). However, the origin and transboundary transmission route of this incursion remain unclear. This study highlights that the two PPR outbreaks in goitered gazelles in the Akesu region of Xinjiang province in 2016 and 2017 may have been caused by another unnoticed incursion. This suggests that goitered gazelles might play an important role in the transboundary transmission of PPR in the region. Goitered gazelle (*G. subgutturosa*) is widely distributed in the Middle East, Central Asia, and West China and Mongolia, where PPRV-associated outbreaks have been widely reported among these ruminants. In 2001, at least 1,500 wild goats and gazelles (*G. subgutturosa*), exhibiting clinical signs similar to those caused by PPRV infection, died in Kavir National Park in Iran (Marashi et al., 2017). During the winters of 2005/2006 and 2008/2009, PPRV infection in rhim gazelle (*G. subgutturosa marica*) and other wild ruminants in the UAE was confirmed (Kinne et al., 2010). The outbreak of PPR in goitered gazelle (*G. subgutturosa*) and other wild ungulates in Mongolia was laboratory-confirmed in December 2016 (Benfield et al., 2021). The cross-border movements of goitered gazelles may have facilitated the transboundary transmission of PPR from the neighboring countries into the Xinjiang province in China. This finding highlights the significance of intensified surveillance for PPR in wildlife in border regions for early detection of a PPR outbreak.

This is the first study to identify anchor mutations and help define PPRV genetic clusters in China. The genomic regions harboring these anchor mutations have been determined, including the 3′ leader region, 5’ UTR of the F gene, and the H and L genes. These anchor mutations can serve as candidate molecular markers for genetic characterization of PPRV circulating in China and help trace the transmission of this virus in the country. The mutations in these genomic regions might be vital to the adaptation of PPRV to the large population of sheep and goats in China. *In vitro* experiments suggest that one mutation in the leader region of the PPRV genome affects virus replication (Eloiflin et al., 2019). The H protein plays a significant role in mediating the entry of PPRV virion particles into the cell and triggering an effective humoral response, which supplements protection against PPRV (Gaur et al., 2024; Rojas et al., 2018). It has been demonstrated that the long M-F UTR of the measles virus controls virus replication and cytopathogenicity (Takeda et al., 2005). The function of F-H UTR remains unclear. The L protein of the *Morbillivirus* genus is a multifunctional catalytic protein that transcribes and replicates the viral genomic RNA. It also performs mRNA capping, methylation, and polyadenylation (Ansari et al., 2019). The effects of these anchor mutations on the viral antigenicity, transmissibility, and pathogenicity of PPRV need to be further investigated.

Our study enhanced the understanding of PPRV evolutionary dynamics. It verified a strong monophyletic relationship among all PPRV genome sequences from China between 2013 and 2024, which share a common ancestor with one strain from the UAE. Through phylogenetic analysis, we identified four different genetic clusters of PPRV in China between 2013 and 2024, with one cluster being the predominant lineage. Moreover, we identified five anchor mutations that define these PPRV genetic clusters in China between 2013 and 2024. This study serves as a valuable example of how genomic epidemiology can be used to trace the evolution of PPRV. Such efforts will play an important role in helping develop effective strategies for PPR control and global eradication initiatives.

## Data Availability

The datasets presented in this study can be found in online repositories. The names of the repository/repositories and accession number(s) can be found in the article/Supplementary material.
