# Supplementary figures and images for: Genomic characterization and evolution analysis of peste des petits ruminants virus in China from 2007 to 2024
Source: Front Microbiol. 2025 Nov 21;16:1697536. doi: 10.3389/fmicb.2025.1697536 (PMC12678265; doi:10.3389/fmicb.2025.1697536)

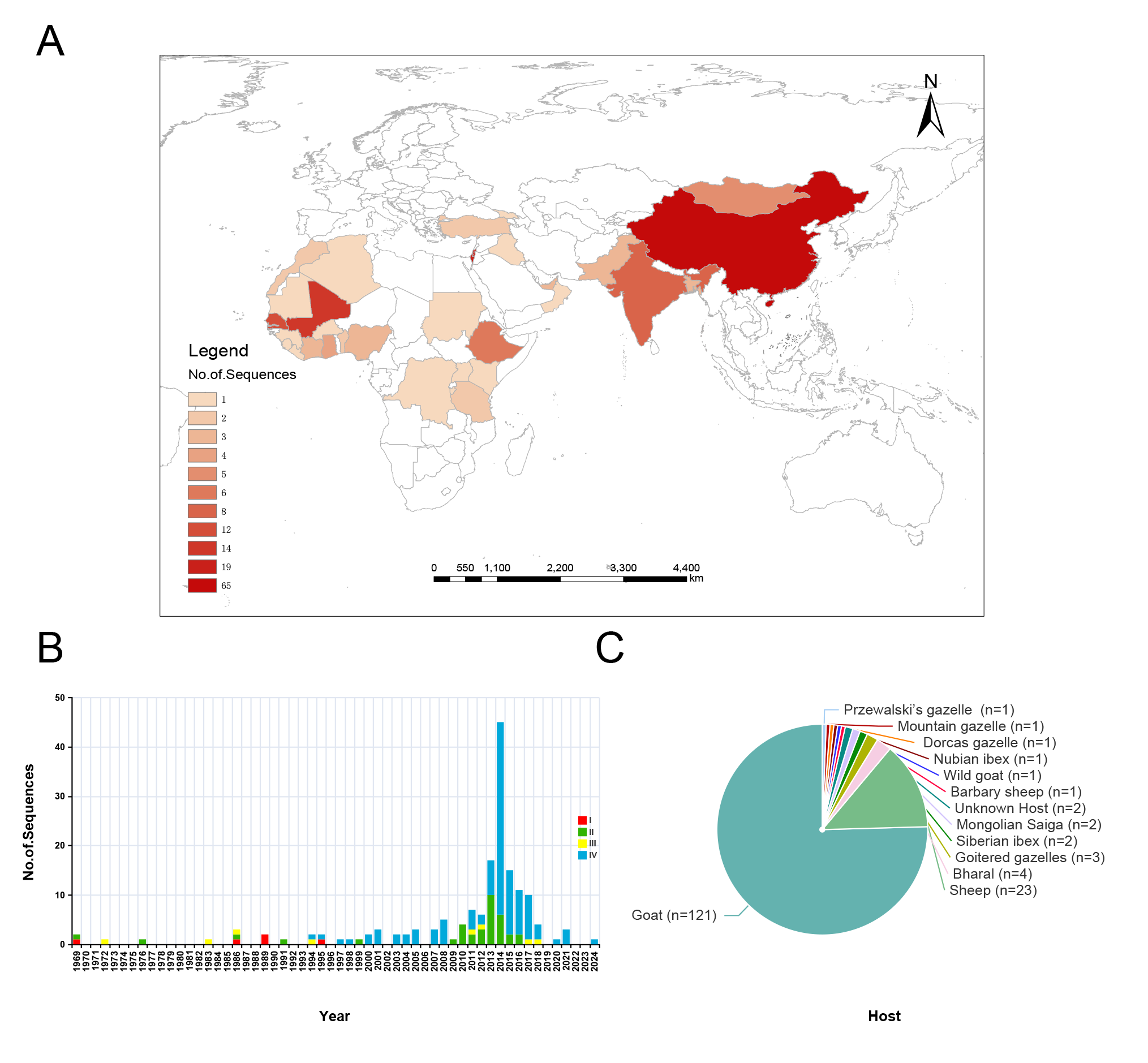

Supplement: Supplementary file 1 [file Image_1.tif]

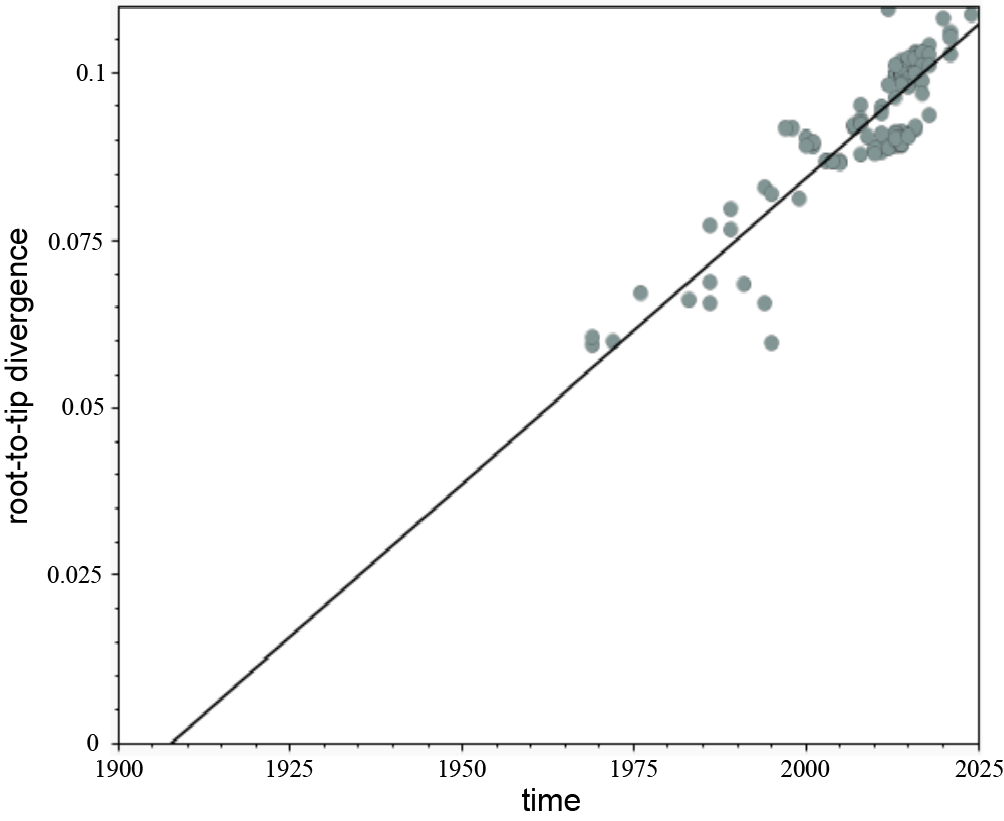

Supplement: Supplementary file 2 [file Image_2.tif]
