# Supplementary material for: Genomic characterization and evolution analysis of peste des petits ruminants virus in China from 2007 to 2024
Source: Front Microbiol. 2025 Nov 21;16:1697536. doi: 10.3389/fmicb.2025.1697536 (PMC12678265; doi:10.3389/fmicb.2025.1697536)
Supplement: Supplementary file 3 [file Table_1.docx]

| GenBank Acc. No. | Strain | Length | Host | Sample | Country | Collection date | Genotype |
| --- | --- | --- | --- | --- | --- | --- | --- |
| PV777992 | China/NX19/2016 | 15954 | Goat | mesenteric lymph node | China | 2016-11-06 | IV |
| PV777993 | China/SX38/2014 | 15954 | Goat | mesenteric lymph node | China | 2014-04-18 | IV |
| PV777994 | China/XJ28/2016 | 15954 | Sibirian ibex | mesenteric lymph node | China | 2016-03-01 | IV |
| PV777995 | China/XJ29/2016 | 15954 | Goitered gazelle | mesenteric lymph node | China | 2016-03-20 | IV |
| PV777996 | China/XJ30/2017 | 15954 | Goitered gazelle | mesenteric lymph node | China | 2017-02-12 | IV |
| PV777997 | China/ZJ8/2014 | 15954 | Goat | mesenteric lymph node | China | 2014-10-11 | IV |
| PV777998 | China/ZJ39/2014 | 15954 | Goat | mesenteric lymph node | China | 2014-04-21 | IV |
| PV777999 | China/JS12/2015 | 15954 | Sheep | mesenteric lymph node | China | 2015-04-07 | IV |
| PV778000 | China/JS13/2015 | 15954 | Sheep | mesenteric lymph node | China | 2015-04-07 | IV |
| PV778001 | China/JX6/2014 | 15954 | Sheep | mesenteric lymph node | China | 2014-07-21 | IV |
| PV778002 | China/JX17/2015 | 15954 | Sheep | mesenteric lymph node | China | 2015-03-30 | IV |
| PV778003 | China/JX22/2014 | 15954 | Sheep | mesenteric lymph node | China | 2014-08-07 | IV |
| PV778004 | China/JL42/2014 | 15954 | Goat | mesenteric lymph node | China | 2014-04-07 | IV |
| PV778005 | China/JL47/2014 | 15954 | Goat | mesenteric lymph node | China | 2014-04-06 | IV |
| PV778006 | China/HN0/2017 | 15954 | Goat | mesenteric lymph node | China | 2017-03-05 | IV |
| PV778007 | China/HN15/2015 | 15954 | Sheep | mesenteric lymph node | China | 2015-05-12 | IV |
| PV778008 | China/HN18/2016 | 15954 | Sheep | mesenteric lymph node | China | 2016-10-16 | IV |
| PV778009 | China/HN23/2015 | 15954 | Sheep | mesenteric lymph node | China | 2015-07-09 | IV |
| PV778010 | China/GD31/2014 | 15954 | Goat | mesenteric lymph node | China | 2014-05-15 | IV |
| PV778011 | China/GX2/2016 | 15954 | Goat | mesenteric lymph node | China | 2016-08-22 | IV |
| PV778012 | China/GZ10/2015 | 15954 | Goat | mesenteric lymph node | China | 2015-05-04 | IV |
| PV778013 | China/GZ11/2015 | 15948 | Goat | mesenteric lymph node | China | 2015-09-02 | IV |
| PV778014 | China/GZ20/2016 | 15954 | Goat | mesenteric lymph node | China | 2016-09-08 | IV |
| PV778015 | China/GZ44/2014 | 15954 | Goat | mesenteric lymph node | China | 2014-04-20 | IV |
| PV778016 | China/CQ9/2014 | 15954 | Goat | mesenteric lymph node | China | 2014-08-15 | IV |
| PV778017 | China/AH16/2015 | 15954 | Sheep | mesenteric lymph node | China | 2015-04-09 | IV |
| PV778018 | China/QHHX/2021 | 15954 | Bharal | mesenteric lymph node | China | 2021-01-22 | IV |
| PV778019 | China/XZLS/2021 | 15954 | Bharal | mesenteric lymph node | China | 2021-02-21 | IV |
| PP937138.1 | ChinaXZ2024 | 15954 | Bharal | spleen | China | 2024-02 | IV |
| FJ905304.1 | China/Tibet/Geg/07-30 | 15948 | Goat | / | China | 2007-08 | IV |
| JF939201.1 | China/Tib/07 | 15948 | Goat | / | China | 2007-12 | IV |
| KX421388.1 | China/33/2007 | 15948 | Goat | / | China | 2007-08 | IV |
| JX217850.1 | Tibet/Bharal/2008 | 15948 | Bharal | / | China | 2008-01 | IV |
| KM091959.1 | China/XJYL/2013 | 15954 | Goat | mesenteric lymph node | China | 2013-11-30 | IV |
| KX421384.1 | China/XJ2/2013 | 15954 | Goat | spleen | China | 2013-12-20 | IV |
| KX421385.1 | China/XJ3/2013 | 15954 | Goat | mesenteric lymph node | China | 2013-12-21 | IV |
| KX421386.1 | China/XJ4/2013 | 15954 | Goat | spleen | China | 2013-12-22 | IV |
| KX421387.1 | China/XJ5/2013 | 15954 | Goat | mesenteric lymph node | China | 2013-12-29 | IV |
| KM089830.1 | CH/HNNY/2014 | 15954 | Goat | / | China | 2014-05-08 | IV |
| KM089831.1 | CH/HNZK/2014 | 15957 | Goat | / | China | 2014-05-08 | IV |
| KM089832.1 | CH/HNZM/2014 | 15954 | Goat | / | China | 2014-05-08 | IV |

| KP868655.1 | CH/GDDG/2014 | 15954 | Goat | lung and fecal sample | China | 2014-12-05 | IV |
| --- | --- | --- | --- | --- | --- | --- | --- |
| MF443335.1 | ChinaZJ2014 | 15954 | Goat | nasal swab | China | 2014-04-25 | IV |
| MF443336.1 | ChinaYN2014 | 15954 | Goat | spleen | China | 2014-04-01 | IV |
| MF443337.1 | ChinaSX2014 | 15954 | Goat | nasal swab | China | 2014-04-05 | IV |
| MF443338.1 | ChinaSC2014 | 15954 | Goat | nasal swab | China | 2014-06-10 | IV |
| MF443339.1 | ChinaSaX2014 | 15954 | Goat | mesenteric lymph node | China | 2014-04-01 | IV |
| MF443340.1 | ChinaNX2014 | 15954 | Sheep | mesenteric lymph node | China | 2014-02-17 | IV |
| MF443341.1 | ChinaLN2014 | 15954 | Goat | mesenteric lymph node | China | 2014-03-17 | IV |
| MF443342.1 | ChinaJX2014 | 15954 | Goat | mesenteric lymph node | China | 2014-04-01 | IV |
| MF443343.1 | ChinaJS2014 | 15954 | Goat | mesenteric lymph node | China | 2014-04-02 | IV |
| MF443344.1 | ChinaJL2014 | 15954 | Sheep | nasal swab | China | 2014-04-01 | IV |
| MF443345.1 | ChinaHN2014 | 15954 | Goat | mesenteric lymph node | China | 2014-04-25 | IV |
| MF443346.1 | ChinaHLJ2014 | 15954 | Goat | nasal swab | China | 2014-03-31 | IV |
| MF443347.1 | ChinaHeN2014 | 15954 | Goat | nasal swab | China | 2014-04-03 | IV |
| MF443348.1 | ChinaHB2014 | 15954 | Goat | mesenteric lymph node | China | 2014-04-03 | IV |
| MF443349.1 | ChinaGZ2014 | 15954 | Goat | nasal swab | China | 2014-04-21 | IV |
| MF443350.1 | ChinaGX2014 | 15954 | Goat | mesenteric lymph node | China | 2014-04-16 | IV |
| MF443351.1 | ChinaGS2014 | 15954 | Goat | mesenteric lymph node | China | 2014-01-22 | IV |
| MF443352.1 | ChinaGD2014 | 15954 | Goat | mesenteric lymph node | China | 2014-05-15 | IV |
| MF443353.1 | ChinaCQ2014 | 15954 | Goat | mesenteric lymph node | China | 2014-03-30 | IV |
| MF443354.1 | ChinaAH2014 | 15954 | Goat | spleen | China | 2014-04-03 | IV |
| KX354359.1 | PPRV-FY | 15948 | Goat | / | China | 2015-12-15 | IV |
| MN121838.1 | ChinaGS2018 | 15954 | Przewalski’s gazelle | / | China | 2018-12 | IV |
| MW344288.1 | ChinaSX2020 | 15954 | Goat | tissue | China | 2020-07 | IV |
| AJ849636.2 | / | 15948 | Sheep | / | Turkey | / | IV |
| EU267273.1 | ICV89 | 15948 | Goat | / | Cote d'Ivoire | / | I |
| EU267274.1 | Ng76/1 | 15948 | Goat | / | Nigeria | / | II |
| KC594074.1 | Morocco 2008 | 15948 | Goat | mesenteric lymph node | Morocco | / | IV |
| KJ466104.1 | Ghana/NK1/2010 | 15948 | Sheep | lung | Ghana | / | II |
| KM212177.1 | SnDk11I13 | 15948 | Goat | eye swab | Senegal | 2013-03-11 | II |
| KJ867540.1 | Ethiopia 1994 | 15948 | Goat | / | Ethiopia | / | III |
| KJ867543.1 | Uganda 2012 | 15948 | Goat | / | Uganda | / | III |
| KJ867544.1 | Oman 1983 | 15948 | Goat | / | Oman | / | III |
| KJ867545.1 | UAE 1986 | 15948 | Dorcas gazelle | / | United Arab Emirates | / | III |
| KM463083.1 | KN5/2011 | 15948 | Goat | lung | Kenya | 2011-05 | III |
| KP789375.1 | E32/1969 | 15948 | Goat | / | Senegal | 1969-09-03 | I |
| KR140086.1 | Izatnagar/94 | 15948 | Goat | / | India | / | IV |
| KT270355.1 | IND/TN/GIN/2014/01 | 15942 | Goat | nasal swab | India | 2014-09-25 | IV |
| KR781449.1 | Benin/10/2011 | 15948 | Sheep | buccal swab | Benin | 2011-05-21 | II |
| KR781450.1 | Benin/B1/1969 | 15948 | Goat | lymph node | Benin | / | II |
| KR781451.1 | CIV/01P/2009 | 15948 | Goat | lung | Cote d'Ivoire | 2009-07 | II |
| KT860063.1 | IND/TN/VM/2014/02 | 15948 | Goat | nasal swab | India | 2014-11-23 | IV |
| KT860064.1 | IND/TN/VEL/2015/03 | 15948 | Sheep | nasal swab | India | 2015-01-05 | IV |

| KT860065.1 | IND/TN/ED/2015/04 | 15948 | Sheep | nasal swab | India | 2015-07-10 | IV |
| --- | --- | --- | --- | --- | --- | --- | --- |
| KR828813.1 | NGYO2013-2162 | 15948 | Goat | / | Nigeria | 2013-02-15 | IV |
| KU236379.1 | Lib/2015 | 15948 | Goat | lung | Liberia | 2015-07-08 | II |
| KX033350.1 | IND/Delhi/2016/05 | 15948 | Goat | nasal swab | India | 2016-01-27 | IV |
| KY888168.1 | PPRV/Mongolia/9/2016 | 15954 | Goat | nasal swab | Mongolia | 2016-09 | IV |
| KY885100.1 | S15 | 15948 | Goat | total blood | Algeria | 2015-11-11 | IV |
| MF737202.1 | Georgia/Tbilisi/2016 | 15948 | Sheep | lung | Georgia | 2016-01-14 | IV |
| MF741712.1 | PPRV/Sierra Leone/048/2011 | 15948 | Goat | lung | Sierra Leone | 2011-12-17 | II |
| MG581412.1 | PPRV/Bangladesh/BD2/2008 | 15948 | Goat | pooled tissue | Bangladesh | 2008-05 | IV |
| MN657232.1 | Turkey/Central_Anatolia/2018 | 15948 | Sheep | lung | Turkey | 2018-09 | IV |
| MK408669.1 | Kurdistan/2011 | 15916 | Wild goat | / | Iraq | 2011-02 | IV |
| MZ322753.1 | Tanzania/Momba/2018 | 15948 | Goat | nasal swab | Tanzania | / | III |
| MZ061719.1 | PPRV/saiga3/Mongolia/2017-01 | 15954 | Mongolian Saiga | / | Mongolia | 2017-01 | IV |
| MZ061720.1 | PPRV/saiga4/Mongolia/2017-01 | 15954 | Mongolian Saiga | / | Mongolia | 2017-01 | IV |
| MZ061721.1 | PPRV/Siberian_ibex/Mongolia/2017-01 | 15932 | Sibirian ibex | / | Mongolia | 2017-01 | IV |
| MZ061722.1 | PPRV/Goitered_gazelle/Mongolia/2017-01 | 15953 | Goitered gazelle | / | Mongolia | 2017-01 | IV |
| OL310685.1 | PPRV/DRC/Tshela/27/2012 | 15948 | Goat | / | Democratic Republic of the Congo | 2012-03 | IV |
| OL310687.1 | PPRV/PPRV/Israel-2536/Hebron/1997 | 15948 | Sheep | lung | Israel | / | IV |
| OL310688.1 | PPRV/Israel-4522/Tzora/1998 | 15948 | Goat | lung | Israel | / | IV |
| OL310689.1 | PPRV/Israel-5236/Ofer/2000 | 15948 | Sheep | lung | Israel | / | IV |
| OL310690.1 | PPRV/Israel-7161/Kseifa/2001 | 15948 | Goat | lung | Israel | / | IV |
| OL310691.1 | PPRV/Israel-5921/Nazareth/2001 | 15948 | Sheep | lung | Israel | / | IV |
| OL310692.1 | PPRV/Israel-6586/Zarzir/2001 | 15948 | Sheep | lung | Israel | / | IV |
| OL310693.1 | PPRV/Israel-2105/Zarzir/2003 | 15948 | Sheep | lung | Israel | / | IV |
| OL310694.1 | PPRV/Israel-2233/Beir-El-Makhsour/2003 | 15948 | Sheep | swab | Israel | / | IV |
| OL310695.1 | PPRV/Israel-1277/Jordan-Valley/2004 | 15948 | Sheep | lung | Israel | / | IV |
| OL310696.1 | PPRV/Israel-1012/Tel-Arad/2004 | 15948 | Goat | lung | Israel | / | IV |
| OL310697.1 | PPRV/Israel-1031/Deir-el-Assad/2005 | 15948 | Sheep | lung | Israel | / | IV |
| OL310698.1 | PPRV/Israel-1034/Deir-el-Assad/2005 | 15948 | Sheep | lung | Israel | / | IV |
| OL310699.1 | PPRV/Israel-1251/Netua/2005 | 15948 | Sheep | lung | Israel | / | IV |
| OL310700.1 | PPRV/Israel-1483/Atauna/2008 | 15948 | Goat | lung | Israel | / | IV |
| OL310701.1 | PPRV/Israel-1921/Goren/2011 | 15948 | Goat | lung | Israel | / | IV |
| OL310702.1 | PPRV/Israel-1947/Goren/2011 | 15948 | Goat | lung | Israel | / | IV |
| OL310703.1 | PPRV/Israel-1192/Rahat/2012 | 15948 | Sheep | lung | Israel | / | IV |
| OL310704.1 | PPRV/Israel-1571/Um-El-Fahem/2014 | 15948 | Sheep | lung | Israel | / | IV |
| OK274214.1 | PPRV/Bangladesh/BD17/2017 | 15948 | Goat | Organ | Bangladesh | / | IV |
| OL741725.1 | PPRV/Morocco/2008/CIRAD | 15948 | Unknown Host | / | Morocco | / | IV |
| ON110960.1 | PPRV/Ethiopia/Habru/2014 | 15948 | Goat | / | Ethiopia | / | IV |
| OL741724.2 | PPRV/Cote_dIvoire/1989/CIRAD | 15948 | Unknown Host | / | Cote d'Ivoire | / | I |
| OR286474.1 | PPRV/Senegal/Dakar/1994 | 15948 | Goat | lung | Senegal | 1995-02 | I |
| OR286475.1 | PPRV/BurkinaFaso/Ouagadoudou/1988 | 15948 | Goat | lung | BurkinaFaso | 1986-06-19 | I |
| OR286476.1 | PPRV/Senegal/Nguekhokh/2/2010 | 15948 | Goat | / | Senegal | 2010-04-25 | II |
| OR286477.1 | PPRV/Senegal/Ngairing/9/2010 | 15948 | Goat | ocular swab | Senegal | 2010-04-28 | II |

| OR286478.1 | PPRV/Mauritania/Tarza/2012 | 15948 | Sheep | ocular swab | Mauritania | / | II |
| --- | --- | --- | --- | --- | --- | --- | --- |
| OR286479.1 | PPRV/Guinea/Dalaba/2013 | 15948 | Goat | ocular swab | Guinea | / | II |
| OR286480.1 | PPRV/Mali/Kolondieba/4/2013 | 15948 | Goat | ocular swab | Mali | 2013-07-27 | II |
| OR286482.1 | PPRV/Mali/Kolondieba/18/2013 | 15948 | Goat | ocular swab | Mali | 2013-07-27 | II |
| OR286483.1 | PPRV/Senegal/Pakour/2/2013 | 15948 | Goat | lung | Senegal | 2013-03-08 | II |
| OR286484.1 | PPRV/Mali/Sagabari/10/2014 | 15948 | Goat | lung | Mali | 2013-07-10 | II |
| OR286485.1 | PPRV/Senegal/SakhMecke/3/2012 | 15948 | Goat | ocular swab | Senegal | 2012-05-21 | II |
| OR286486.1 | PPRV/Senegal/MbourJoal/2/2013 | 15948 | Goat | lung | Senegal | 2013-01-05 | II |
| OR286487.1 | PPRV/Senegal/GayeMecke/1/2013 | 15948 | Goat | lung | Senegal | 2013-01-05 | II |
| OR286488.1 | PPRV/Senegal/Soum/2/2012 | 15948 | Goat | ocular swab | Senegal | 2012-03-08 | II |
| OR286489.1 | PPRV/Senegal/Ngairing/1/2010 | 15948 | Goat | ocular swab | Senegal | 2010-04-28 | II |
| OR286490.1 | PPRV/Mali/Segou/3/2014 | 15948 | Goat | ocular swab | Mali | / | II |
| OR286491.1 | PPRV/Mali/Samako/9/2014 | 15948 | Goat | ocular swab | Mali | / | II |
| OR286492.1 | PPRV/Mali/Samako/10/2014 | 15948 | Goat | ocular swab | Mali | / | II |
| OR286493.1 | PPRV/Mali/Samako/12/2014 | 15948 | Goat | ocular swab | Mali | / | II |
| OR286494.1 | PPRV/Mali/Samako/13/2014 | 15948 | Goat | ocular swab | Mali | / | II |
| OR286495.1 | PPRV/Mali/Tousseguela/14/2014 | 15948 | Goat | ocular swab | Mali | / | II |
| OR286496.1 | PPRV/Mali/Kolondieba/5/2013 | 15948 | Goat | ocular swab | Mali | 2013-07-27 | II |
| OR286497.1 | PPRV/Mali/Kolondieba/1/2013 | 15948 | Goat | ocular swab | Mali | 2013-07-27 | II |
| OR286498.1 | PPRV/Ghana/AttaBagbe/2014 | 15948 | Goat | lung | Ghana | 2014-03-03 | II |
| OR286499.1 | PPRV/Senegal/Kedougou/31/2016 | 15948 | Goat | ocular swab | Senegal | / | II |
| OR286500.1 | PPRV/Mali/Bamako/1999 | 15948 | Goat | lung | Mali | / | II |
| OR286501.1 | PPRV/Ghana/Accra/1976 | 15948 | Goat | lung | Ghana | 1991-04-26 | II |
| OR286502.1 | PPRV/Ghana/Accra/1978 | 15948 | Goat | lung | Ghana | 1986-06-19 | II |
| OR286503.1 | PPRV/Mali/Kayes/39b/2016 | 15948 | Goat | ocular swab | Mali | 2016-04-06 | II |
| OR286504.1 | PPRV/India/Calcutta/1995 | 15948 | Goat | lymph node | India | 1995-03-22 | IV |
| OR286505.1 | PPRV/Sudan/Sinar/1972 | 15948 | Goat | lymph node | Sudan | / | III |
| MK991798.1 | SRMV/Ethiopia/2011 | 15948 | Goat | lung | Ethiopia | 2011-05 | IV |
| MK991799.1 | SRMV/Ethiopia/2014 | 15948 | Goat | / | Ethiopia | / | IV |
| MK991800.1 | SRMV/Ethiopia/2017 | 15948 | Goat | lung | Ethiopia | / | IV |
| MN369543.1 | SMRV/IND/2013/V242.5/Shahjadpur | 15929 | Goat | / | India | 2013-02-14 | IV |
| MN369542.1 | SMRV/UAE/2018/V135/Dubai | 15954 | Mountain gazelle | / | United Arab Emirates | 2018-08-14 | IV |
| OM867572.1 | V346.2 | 15948 | Barbary sheep | tissue | United Arab Emirates | / | IV |
| MF678816.1 | PPRV/KVI/2017 | 15927 | Nubian ibex | lung and gut | Israel | 2017-01 | IV |
| KY967610.1 | SRMV/Layyah/UVAS/Pak/2015 | 15948 | Sheep | / | Pakistan | / | IV |
| MK686066.1 | B3 | 15948 | Goat | lung | Burundi | 2017-12 | III |
