# Supplementary material for: Genomic characterization and evolution analysis of peste des petits ruminants virus in China from 2007 to 2024
Source: Front Microbiol. 2025 Nov 21;16:1697536. doi: 10.3389/fmicb.2025.1697536 (PMC12678265; doi:10.3389/fmicb.2025.1697536)
Supplement: Supplementary file 4 [file Table_2.docx]

Table S2. Recombination analysis using RDP4

| Recombinant | Major parent | Minor parent | RDP | GENECONV | Detection methods | | | SiSscan | 3Seq |
| --- | --- | --- | --- | --- | --- | --- | --- | --- | --- |
|  |  |  |  |  | Bootscan | Maxchi | Chimaera |  |  |
| KR828814. 1 Nigeria 2012-05-09 | KR781449. 1 Benin 10 2011-05-21 | KR828813. 1 Nigeri3 2013-02-15 | + | + | + | + | + | + | + |
| KJ867541. 1 Ethiopia 2010 | ON110980. 1 Ethiopia Habru 2014 | KC594074. 1 Morocco 2008 | + | + | + | + | + | + | + |
| KY967609. 1 Pakistan Faisalabad 2015 | KY967608. 1 Pakistan Lahore 2015 | KT880085. 1 India 04 2015-07-10 | + | + | + | + | + | + | + |
| OK274213. 1 Bangladesh BD12 2015 | FJ905304. 1 China Tibet 07-30 2007-08 | MG581412. 1 Bangladesh BD2 2008-05 | + | + | - | + | + | + | + |
| OR286481. 1 Mali Kolondieba 6 2013 | OR286495. 1 Mali Tousseguela 14 2014 | OR288480. 1 Mali Kolondieba 4 2013 | + | + | + | + | + | - | + |
| KY967608. 1 Pakistan Lahore 2015 | KY967609. 1 Pakistan Faisalabad 2015 | KT880085. 1 India 04 2015-07-10 | + | + | + | + | + | - | + |
| MW960272. 1 Tanzania Ngorongoro 2016 | KJ867543. 1 Uganda 2012 | MZ322753.1 Tanzania Momba 2018 | + | + | + | + | + | + | + |
| KR261605. 1 India 2014-09-16 | KT270355. 1 India 2014-09-25 | OL310690. 1 lsrael Kseifa 2001 | + | + | - | + | - | + | + |
