# Supplementary material for: Genomic characterization and evolution analysis of peste des petits ruminants virus in China from 2007 to 2024
Source: Front Microbiol. 2025 Nov 21;16:1697536. doi: 10.3389/fmicb.2025.1697536 (PMC12678265; doi:10.3389/fmicb.2025.1697536)
Supplement: Supplementary file 5 [file Table_3.docx]

Table S3. List of amino acid positions identified as evolving under positive selection.

| Protein | AA mutation | MEME | FUBAR | FEL | SLAC |
| --- | --- | --- | --- | --- | --- |
| H | P438L | 438 | / | 438 | / |
| L | A246G | 246 | 246 (0.95) | 246 | / |
|  | Y336H | 336 | / | 336 | / |
|  | P647L | 647 | / | 647 | / |
|  | G1122A | 1122 | / | 1122 | / |
|  | L1708S | 1708 | / | 1708 | / |

The default threshold of significance (P < 0.1) was used for MEME, SLAC and FEL. Posterior probability of 0.95 was used for FUBAR. Posterior probability was given in parentheses. ‘/’ indicates no positively selected sites was identified.
