# Supplementary material for: Genomic characterization and evolution analysis of peste des petits ruminants virus in China from 2007 to 2024
Source: Front Microbiol. 2025 Nov 21;16:1697536. doi: 10.3389/fmicb.2025.1697536 (PMC12678265; doi:10.3389/fmicb.2025.1697536)
Supplement: Supplementary file 6 [file Table_4.docx]

Table S4. Estimation of mean evolutionary rates of PPRV in previous studies and this study.

| Data set | Time Period | Mean Evolutionary rate | 95% HPD Interval | Reference |
| --- | --- | --- | --- | --- |
| 39 | 1969-2015 | 9.22×10^-4^ | 6.21×10^-4^-1.26×10^-3^ | [14] |
| 81 | 1969-2018 | 9.22×10^-4^ | 6.78×10^-4^-1.17×10^-3^ | [11] |
| 103 | 1969-2018 | 8.098×10^-4^ | 5.867×10^-4^-9.056×10^-4^ | [10] |
| 136 | 1969-2021 | 6.04×10^-4^ | 4.68×10^-4^-7.53×10^-4^ | [12] |
| 163 | 1969-2024 | 6.70×10^-4^ | 5.63×10^-4^-7.87×10^-4^ | This study |
