# Supplementary material for: Genomic characterization and evolution analysis of peste des petits ruminants virus in China from 2007 to 2024
Source: Front Microbiol. 2025 Nov 21;16:1697536. doi: 10.3389/fmicb.2025.1697536 (PMC12678265; doi:10.3389/fmicb.2025.1697536)
Supplement: Supplementary file 7 [file Table_5.docx]

Table S5. TMRCA estimate for lineage IV PPRV in previous studies and this study.

| Data set | Time Period | TMRCA | 95% HPD Interval | Reference |
| --- | --- | --- | --- | --- |
| 39 | 1994-2015 | 1968 | 1880-1987 | [14] |
| 66 | 1994-2018 | 1975 | 1961-1986 | [11] |
| 85 | 1994-2018 | 1967 | 1948-1981 | [10] |
| 86 | 1994-2021 | 1963 | 1938-1980 | [12] |
| 115 | 1994-2024 | 1966 | 1937-1984 | This study |
